# Transient Erythema Elevatum Diutinum Associated With HIV Viremia

**DOI:** 10.7759/cureus.40858

**Published:** 2023-06-23

**Authors:** Hannah Rashdan, Helen Schafer, Ashley D Lundgren, Kaylee O'Connor, Brett Keeling

**Affiliations:** 1 Dermatology, University of Texas at Austin Dell Medical School, Austin, USA

**Keywords:** dermatopathology, anti-retroviral therapy, vasculitis, hiv/aids, erythema elevatum diutinum

## Abstract

Erythema elevatum diutinum (EED) is a rare cutaneous small vessel vasculitis of unknown etiology. It is thought to be due to immune complex deposition in small vessels, resulting in complement fixation and subsequent inflammation. EED classically presents with asymptomatic, symmetric, red-brown to purple papules, plaques, and nodules overlying extensor surfaces with a lapsing-remitting course that typically resolves within five to 10 years. We discuss the case of a 47-year-old male with HIV and a new history of EED presenting after several days of missed antiretroviral medications and resolved with improved compliance with antiretroviral medications. A 47-year-old male presented with a four-week history of mildly tender violaceous plaques and nodules on the dorsal feet and posterior heels bilaterally. Medical history was significant for HIV that was well-controlled on antiretrovirals although the patient had missed two days of therapy. A punch biopsy of the lesion demonstrated leukocytoclastic vasculitis with dense dermal mixed infiltrate consisting of histiocytes, neutrophils, and eosinophils. Laboratory findings revealed the presence of HIV RNA. Prior to the initiation of Dapsone therapy, the patient’s eruption cleared entirely within a month solely by restarting his antiretroviral therapy, for which he continues to remain disease-free. EED is a rare, chronic leukocytoclastic vasculitis with a poorly understood etiology. Treatment is typically aimed at treating underlying systemic disease, however, treatment of EED with Dapsone is typically first-line.

## Introduction

Erythema elevatum diutinum (EED) is a rare chronic leukocytoclastic vasculitis of unknown etiology [1]. Leukocytoclastic vasculitis is a cutaneous, small vessel vasculitis of the dermal capillaries and venules [2]. While the exact mechanism is unknown, EED is thought to be due to immune-complex deposition in dermal blood vessels, resulting in complement fixation and subsequent inflammation [1]. The inflammatory response of EED is secondary to an activation of the complement cascade via IL-8, with neutrophil chemotaxis releasing lysozymes, collagenases, myeloperoxidases, and hydrolases. These induce fibrin deposition and cholesterol crystals in the small vessels [3].

EED classically presents with asymptomatic, symmetric red-brown to purple papules, plaques, and nodules overlying extensor surfaces with a lapsing-remitting course that typically resolves within five to 10 years [4]. The differential diagnosis for EED includes Sweet’s syndrome, granuloma annulare, and Kaposi sarcoma, among others. These may be differentiated by clinical exam and by histologic analysis, with EED demonstrating leukocytoclastic vasculitis with angiocentric histiocytes, neutrophils, and eosinophils on acral skin [2,5].

EED is known to be associated with underlying disease. These include hematologic, rheumatologic, and infectious causes. Some of the more commonly reported hematologic disorders include paraproteinemia and myeloproliferative and dysplastic disorders. EED is known to be associated with rheumatologic diseases such as celiac disease, inflammatory bowel disease, systemic lupus erythematosus, and rheumatoid arthritis. Finally, infections such as syphilis and viral hepatitis are known to be associated with EED. Associations between EED and systemic diseases are likely due to circulating immune complexes, leading to complement deposition and leukocytoclastic vasculitis [4,6,7].

While a handful of cases report an association between HIV and EED, these are rare, with EED typically occurring months after untreated HIV [8-10]. We discuss the case of a 47-year-old male with HIV and a four-week history of EED presenting after several days of missed antiretroviral medications and resolved with improved compliance with antiretroviral medications.

## Case presentation

**Figure 1:**
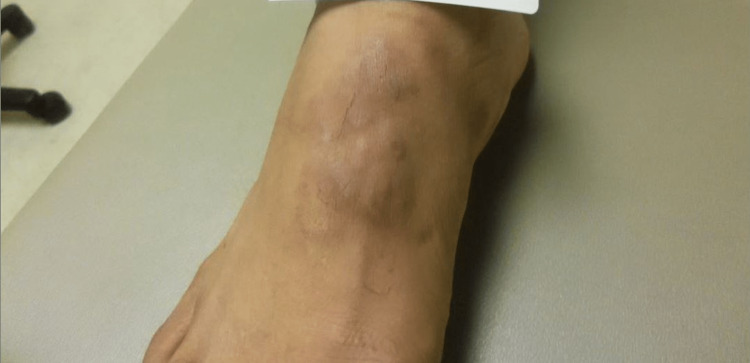
Violaceous plaques and nodules on the right dorsal foot

A 47-year-old male presented with a four-week history of mildly tender violaceous plaques and nodules on the dorsal feet and posterior heels bilaterally (Figure 1). The lesions were asymptomatic except for tenderness with friction contact with footwear. Medical history was significant for HIV, which was well-controlled on an antiretroviral regimen, although the patient had missed medications for the past two days. He had no other significant past medical history and was not taking other medication. He had no other cutaneous findings and a review of systems was within normal limits.

**Figure 2:**
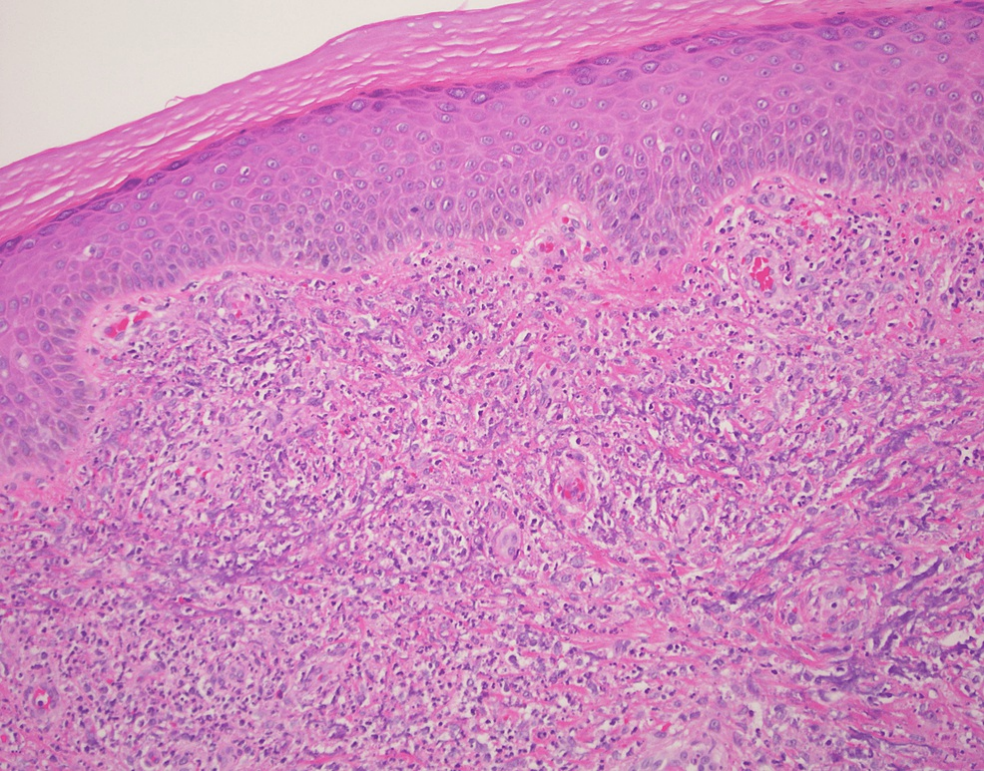
Histopathologic view of the right dorsal foot punch biopsy demonstrating mixed dermal infiltrates with histiocytes, eosinophils, lymphocytes, and leukocytoclasis (Hematoxylin & Eosin stained, x10)

A 4-mm punch biopsy from the right dorsal foot was obtained, demonstrating leukocytoclastic vasculitis with dense, dermal mixed infiltrate consisting of histiocytes, neutrophils, and eosinophils (Figure 2). Periodic acid-Schiff (PAS) and fite stains were negative.

Laboratory findings revealed a normal complete blood count and basic metabolic panel, elevated alanine aminotransferase at 27 (normal range 8-10 U/L), thyroid stimulating hormone at 0.51 uIU/mL (normal range 0.5-5.0 uIU/mL), and the presence of HIV RNA (46 copies/mL). Given this finding, a T-lymphocyte differential was ordered and revealed normal CD3+, CD4+, and CD8+ cell counts (Table 1). Laboratory markers for underlying autoimmune or other infectious causes were also negative (Table 2).

**Table 1:** Initial presentation of T-lymphocyte differential

	T-lymphocyte, %	Absolute T-Lymphocyte Count, cells/μL
CD3	76	4267
CD4	30	1678
CD8	52	2929

**Table 2:** Laboratory panel taken at initial presentation to diagnose underlying systemic disease, including rheumatologic diseases, often associated with EED * Includes antibodies to detect previous infection with hepatitis A, hepatitis B, or hepatitis C ** Includes ANCA and proteins myeloperoxidase (MPO) and proteinase 3 (PR3) EED: erythema elevatum diutinum; ANCA: antineutrophil cytoplasmic antibodies

Laboratory Assessment	Result
Hepatitis panel*	Negative
Antineutrophil cytoplasmic antibodies (ANCA) panel**	Negative
Rheumatoid factor (RF)	Negative
Thyroid stimulating hormone (TSH)	0.51 U/mL (Normal 0.5 to 5.0 mlU/L)
Quantiferon gold	Negative
Serum protein electrophoresis (SPEP)	Normal
Rapid plasma reagin (RPR)	Negative

Glucose-6-phosphate dehydrogenase levels were drawn in preparation for starting the patient on oral Dapsone. The patient also restarted antiretroviral therapy for his previously diagnosed HIV. Prior to the initiation of Dapsone, the patient’s eruption cleared entirely within a month. Repeat levels of the patient’s HIV RNA load were undetectable. The patient has remained disease-free for 17 months, solely with adherence to his antiretroviral regimen with no recurrence of EED.

## Discussion

We report a case of EED isolated to the feet that occurred in the setting of transient HIV viremia and a normal CD4+ count after two days of missed antiretrovirals. The diagnosis of EED is both clinical and histopathological. In the early stage of the disease, leukocytoclastic vasculitis presents with polymorphonuclear cells, macrophages, and histocytes in the dermis. In the late stage of EED, granulation tissue, fibrosis, vascular proliferation, lymphohistiocytic inflammatory infiltrate, and areas of neutrophils with leukocytoclastic may be seen [3]. While these features are distinguishing, histopathological diagnosis of a type of leukocytoclastic vasculitis, such as EED, may be challenging due to the evolving nature of these abnormalities [11]. Additionally, the inflammatory infiltrate seen in the early stage of EED may be seen in other conditions in an HIV-positive patient, such as Kaposi sarcoma and bacillary angiomatosis, and must be differentiated both clinically and histopathologically [3].

EED in HIV patients tends to display extensive and advanced involvement with nodular, fibrotic, and bulky lesions on the palms and soles, sometimes with blistering and ulceration. Older lesions may show onion-skin fibrosis and extracellular cholesterol clefts from chronic erythrocyte extravasation [9,10]. Dapsone is the most effective treatment, particularly in the setting of HIV [4]. However, other agents, such as topical, intralesional or oral steroids, antimicrobials (sulfonamides, nicotinamide), and colchicine have also been successful [4,6]. Local surgical excision has even been reported as beneficial for the localized fibrotic nodules of EED [6].

The timely diagnosis and treatment of EED are important, as this condition points to underlying systemic conditions, and older lesions may be less responsive to therapeutic interventions due to the nodular fibrosis characteristic of long-standing EED. However, even with treatment, the recurrence rate of EED is high, with complete resolution of the disease not always possible [12,13]. Treatment of underlying triggering factors, such as systemic disease, may provide the highest chance of success in the prevention of relapse of EED [6]. Additionally, for patients without a known coexisting autoimmune, hematologic, and/or infectious disease, a close follow-up is necessary to monitor for its development, considering EED’s association with severe systemic conditions [14,15]. Finally, while EED is commonly asymptomatic, the presenting nodular fibrosis can be disfiguring, and treatment as early in the course of the disease as possible is essential [13].

## Conclusions

This case demonstrates a unique example of EED with an underlying HIV diagnosis and transient viremia days after interruption of antiretroviral therapy. The patient’s cutaneous EED resolved with antiretroviral therapy alone, demonstrating the importance of treating underlying pathology when suspecting EED.
